# Clinical Outcomes of Isolated Abnormal Umbilical Artery Doppler Findings in Normally Grown Fetuses: A Retrospective Cohort Study

**DOI:** 10.7759/cureus.115868

**Published:** 2026-09-06

**Authors:** Ei Ei Phyo Naing, Mouli Nandi, Rana Mondal, Jodie Lam

**Affiliations:** 1 Obstetrics and Gynaecology, Luton and Dunstable University Hospital, Luton, GBR; 2 Obstetrics and Gynaecology, Luton and Dunstable University Hospital NHS Foundation Trust, Luton, GBR

**Keywords:** fetal growth restriction, fetal surveillance, placental dysfunction, small for gestational age, umbilical artery doppler

## Abstract

Background: Umbilical artery Doppler (UAD) is widely used to assess placental function in pregnancies complicated by fetal growth restriction (FGR), but the clinical significance of an abnormal UAD in a fetus that is normally grown at the time of diagnosis is less certain. This study describes the subsequent Doppler course and pregnancy outcomes of such cases.

Materials and methods: We performed a retrospective cohort study at Luton and Dunstable University Hospital of pregnancies with abnormal UAD identified between January and December 2025. Abnormal UAD was defined as an umbilical artery pulsatility index above the 95th centile for gestational age, with absent or reversed end-diastolic flow also classified as abnormal. Normal growth was defined as AGA (appropriate for gestational age), with an EFW (estimated fetal weight) and/or AC (abdominal circumference) between the 10th and 90th centiles; pregnancies with established SGA (small for gestational age) or FGR at the index assessment were excluded. Electronic records were reviewed for Doppler progression, subsequent SGA/FGR, pre-eclampsia, mode of delivery, and neonatal outcomes. Descriptive statistics were used.

Results: During the study period, 1,372 UAD examinations were performed, and abnormal UAD was identified in 168 pregnancies. Fourteen pregnancies had incomplete follow-up, leaving 154 for analysis; 88/154 (57.1%) had normal fetal growth at the index abnormal UAD and formed the study cohort. UAD normalised on follow-up in 43/88 (48.9%) and remained abnormal in 45/88 (51.1%). Among pregnancies with persistent abnormal UAD, 11/45 (24.4%) subsequently developed SGA or FGR. Three of 88 pregnancies (3.4%) developed pre-eclampsia. The mean time to Doppler normalisation was 1.78 weeks. Among the persistent abnormal UAD group, 22/45 (48.9%) underwent induction of labour and 53/88 (60.2%) had caesarean births. Eight neonates (9.1%) were admitted to the neonatal intensive care unit. No stillbirths or early neonatal deaths were observed.

Conclusion: Approximately half of isolated abnormal UAD findings in normally grown fetuses normalised on follow-up, while half persisted. Subsequent SGA/FGR occurred in 24.4% of the persistent group. These descriptive findings support continued surveillance after an isolated abnormal UAD and justify prospective studies with a comparator group and standardised management to define prognostic significance.

## Introduction

Umbilical artery Doppler (UAD) assessment is an established tool for evaluating fetoplacental circulation and is routinely incorporated into the assessment and surveillance of pregnancies complicated by fetal growth restriction (FGR) [1-3]. Abnormal findings, including an elevated pulsatility index (PI) and absent or reversed end-diastolic flow, are associated with placental dysfunction and adverse perinatal outcomes in growth-restricted fetuses [1,3].

In contrast, the significance of an abnormal UAD when fetal growth is normal at the time of assessment is less well defined. At our institution, UAD is routinely assessed as part of fetal growth scans, including when fetal biometry is appropriate for gestational age (AGA). Consequently, abnormal UAD findings may be identified in fetuses with otherwise normal growth. Evidence describing their natural history, subsequent fetal growth, and pregnancy outcome remains limited. Longitudinal studies have demonstrated changes in uterine, umbilical, and fetal cerebral Doppler indices with evolving fetal growth abnormalities [4]. Previous cohort studies have also reported associations between abnormal UAD in apparently normally grown or appropriately grown fetuses and subsequent small for gestational age (SGA), markers of FGR, and neonatal intensive care unit (NICU) admission [5,6]. This study therefore aimed to describe the clinical course and outcomes of pregnancies with isolated abnormal UAD in normally grown fetuses.

## Materials and methods

This retrospective cohort study was conducted at Luton and Dunstable University Hospital and included pregnancies in which an abnormal UAD was identified between January and December 2025. During the study period, 1,372 UAD examinations were performed, including repeat examinations where clinically indicated, and abnormal UAD was identified in 168 pregnancies.

Abnormal UAD was defined as an umbilical artery PI above the 95th centile for gestational age. Absent or reversed end-diastolic flow, where present, was also classified as abnormal. Normal fetal growth was defined as appropriate for gestational age (AGA), with an estimated fetal weight (EFW) and/or abdominal circumference (AC) between the 10th and 90th centiles for gestational age at the index assessment. Pregnancies with established SGA or FGR at the index abnormal UAD were excluded from the normally grown cohort.

Data were extracted from electronic medical records. Variables included gestational age at the index abnormal UAD, subsequent Doppler findings, development of SGA or FGR, pre-eclampsia, mode and indication for delivery, and neonatal outcomes, including NICU admission, stillbirth, and early neonatal death.

Doppler findings were classified as normalised when a subsequent UAD no longer met the study definition of abnormality and as persistent when abnormality remained on follow-up.

Data were analysed using Microsoft Excel (Microsoft Corporation, Redmond, Washington). Descriptive statistics are presented as counts and percentages for categorical variables and as the mean for time to Doppler normalisation. No formal between-group hypothesis testing or multivariable analysis was performed; therefore, the study was intended to describe patterns and outcomes rather than estimate independent associations.

The study was registered and logged with the Clinical Quality, Audit and Effectiveness Department at Luton and Dunstable University Hospital (FORMIC ID: 1393).

## Results

A total of 168 pregnancies with abnormal UAD were identified. Fourteen were excluded because follow-up was incomplete, leaving 154 pregnancies for analysis. Of these, 88/154 (57.1%) had normal fetal growth at the index abnormal UAD and comprised the study cohort. The study population and Doppler outcomes are summarised in Table 1.

**Table 1:** Study population and Doppler outcomes UAD: umbilical artery Doppler

Variable	Value, n or n(%)
Total UAD examinations performed	1,372
Pregnancies with abnormal UAD identified	168
Excluded because of incomplete follow-up	14
Pregnancies remaining for analysis	154
Normal fetal growth at index abnormal UAD	88/154 (57.1%)
UAD normalised on follow-up	43/88 (48.9%)
Persistent abnormal UAD	45/88 (51.1%)

Within the 88 normally grown pregnancies, UAD normalised on follow-up in 43 (48.9%) and remained abnormal in 45 (51.1%). Among the 45 pregnancies with persistent abnormal UAD, 11 (24.4%) subsequently developed SGA or FGR. Three of the 88 pregnancies (3.4%) developed pre-eclampsia. The mean time to UAD normalisation was 1.78 weeks.

Among the 45 pregnancies with persistent abnormal UAD, induction of labour was undertaken in 22 (48.9%). Caesarean birth occurred in 53/88 pregnancies (60.2%).

Eight of 88 neonates (9.1%) required NICU admission. No stillbirths or early neonatal deaths were recorded. Maternal, fetal, delivery, and neonatal outcomes are summarised in Table 2.

**Table 2:** Clinical outcomes of the normally grown cohort SGA: small for gestational age; FGR: fetal growth restriction; UAD: umbilical artery Doppler; NICU: neonatal intensive care unit

Outcome	Value, n (%)	Denominator/note
Subsequent SGA/FGR	11/45 (24.4%)	Persistent abnormal UAD group
Pre-eclampsia	3/88 (3.4%)	Full normally grown cohort
Induction of labour	22/45 (48.9%)	Persistent abnormal UAD group
Caesarean birth	53/88 (60.2%)	Full normally grown cohort
NICU admission	8/88 (9.1%)	Full normally grown cohort
Stillbirth/early neonatal death	0/88 (0%)	Full normally grown cohort

## Discussion

In this single-centre retrospective cohort, approximately half of the isolated abnormal UAD findings in normally grown fetuses normalised on subsequent assessment, while approximately half persisted. Among the 45 pregnancies with persistent abnormal UAD, 11/45 (24.4%) subsequently developed SGA or FGR. These findings suggest that an abnormal UAD in a fetus that is appropriately grown at the time of assessment may represent an early manifestation of placental dysfunction in a subset of pregnancies. However, the descriptive study design and absence of a contemporaneous normal-UAD control group mean that causality or independent prognostic value cannot be established.

UAD reflects downstream resistance within the fetoplacental circulation and has an established role in the assessment and surveillance of pregnancies complicated by FGR [1-3]. Contemporary guidelines recommend serial UAD assessment after FGR has been diagnosed, particularly when increased placental resistance or absent or reversed end-diastolic flow is present [7,8]. However, considerably less evidence is available to guide management when fetal biometry remains AGA despite an abnormal UAD. This creates a clinically important area of uncertainty because an isolated abnormal result may represent transient or measurement-related variation, or an early manifestation of placental dysfunction that precedes detectable impairment of fetal growth.

Our finding that 11/45 (24.4%) pregnancies with persistent abnormal UAD subsequently developed SGA or FGR is consistent with previous studies examining abnormal UAD in normally or appropriately grown fetuses. Al Hamayel et al. reported an association between abnormal UAD in normally grown fetuses and subsequent SGA and NICU admission [5]. Similarly, Mathewlynn et al., in a five-year retrospective cohort of appropriately grown fetuses, found that a raised umbilical artery PI above the 95th centile in the early third trimester was associated with subsequent markers of FGR and an increased risk of SGA at birth [6]. Together with the present findings, these studies support the possibility that increased placental vascular resistance may become detectable before fetal size crosses conventional thresholds used to identify SGA or FGR.

The relationship between fetal size, placental function, and Doppler abnormalities is nevertheless complex. Earlier work evaluating Doppler findings in small and normally grown fetuses demonstrated that the prognostic performance of UAD differed according to fetal growth status [9]. This is important when interpreting the present cohort because evidence derived from established SGA or FGR cannot necessarily be extrapolated directly to an appropriately grown population. Nevertheless, the findings of Al Hamayel et al. and Mathewlynn et al. [5,6], together with our observation of subsequent SGA/FGR among some pregnancies with persistent abnormal UAD, suggest that normal fetal biometry at a single assessment does not necessarily exclude evolving placental dysfunction.

An important finding in our cohort was that UAD abnormalities normalised on follow-up in 43/88 (48.9%) pregnancies, with a mean time to normalisation of 1.78 weeks. This demonstrates that an isolated abnormal UAD does not invariably persist and highlights the potential value of repeat assessment before attributing prognostic significance to a single abnormal measurement. Conversely, subsequent SGA/FGR occurred in 11/45 (24.4%) pregnancies in which the abnormal UAD persisted, supporting continued assessment of fetal growth and Doppler findings when abnormalities remain present. Longitudinal studies in established SGA have demonstrated changes in uterine, umbilical, and fetal cerebral Doppler indices as placental disease evolves [4]. Although these populations differ from the normally grown fetuses included in the present study, they provide a physiological basis for considering Doppler findings as dynamic rather than static measurements.

Delivery intervention was common in our cohort. Among pregnancies with persistent abnormal UAD, 22/45 (48.9%) underwent induction of labour, while caesarean birth occurred in 53/88 (60.2%) of pregnancies overall. These figures should be interpreted cautiously. The abnormal Doppler finding itself may have influenced clinicians' decisions regarding surveillance and the timing or mode of delivery, producing confounding by indication. Therefore, the high intervention rates cannot be assumed to represent adverse consequences of the Doppler abnormality itself. Neonatal outcomes were generally favourable: 8/88 (9.1%) neonates required NICU admission, and there were no stillbirths or early neonatal deaths. The relatively small cohort, however, limits the ability to assess uncommon but clinically important adverse perinatal outcomes.

The findings have potential implications for clinical practice. Current international guidance provides detailed recommendations for surveillance and delivery when abnormal UAD occurs in association with established FGR [7,8,10]. These guidelines recognise UAD as an important component of assessing placental dysfunction and fetal wellbeing, but there is less clarity regarding isolated abnormal UAD in an appropriately grown fetus. The present findings do not support intervention based on a single abnormal measurement. Rather, the high proportion of abnormalities that subsequently normalised, together with the development of SGA/FGR in a subset with persistent abnormality, suggests that repeat Doppler assessment and continued surveillance of fetal growth may be appropriate when an isolated abnormal UAD is identified. However, our study cannot determine the optimal frequency of surveillance or establish thresholds for delivery, and these findings should therefore be considered hypothesis-generating rather than prescriptive.

This study has several limitations. Its retrospective, single-centre design limits generalisability and makes it susceptible to incomplete documentation and selection bias. Fourteen pregnancies were excluded because of incomplete follow-up. There was no contemporaneous control group of normally grown fetuses with normal UAD, formal comparative statistical testing was not undertaken, and potential confounders, including gestational age, maternal comorbidity, and clinical management decisions, were not adjusted for. Detailed analysis of indications for caesarean delivery according to Doppler and fetal growth outcomes was not performed. In addition, the 1,372 UAD examinations included repeat assessments from the same pregnancy and therefore cannot be used as a denominator to estimate pregnancy-level prevalence. The sample size also limits the assessment of uncommon outcomes, such as stillbirth.

Larger prospective studies using a standardised definition of isolated abnormal UAD, prespecified follow-up intervals, an appropriately grown control group with normal UAD, and adjustment for relevant maternal and fetal confounders are needed. Such studies could determine whether persistence, severity, or gestational age at first abnormal UAD independently predicts subsequent FGR or adverse perinatal outcomes and could inform more consistent surveillance and delivery strategies.

## Conclusions

In this retrospective cohort of normally grown fetuses with an isolated abnormal UAD, 43/88 (48.9%) of Doppler abnormalities normalised on follow-up and 45/88 (51.1%) persisted. Among the persistent group, 11/45 (24.4%) subsequently developed SGA or FGR. These findings support continued surveillance after an isolated abnormal UAD, but because the study was descriptive and lacked a normal-UAD comparator group, they do not establish an independent association with adverse outcomes. Larger prospective studies are needed to define prognostic significance and guide standardised management.
